# Protein/Arabinoxylans Gels: Effect of Mass Ratio on the Rheological, Microstructural and Diffusional Characteristics

**DOI:** 10.3390/ijms151019106

**Published:** 2014-10-21

**Authors:** Claudia M. Berlanga-Reyes, Elizabeth Carvajal-Millan, Kevin B. Hicks, Madhav P. Yadav, Agustín Rascón-Chu, Jaime Lizardi-Mendoza, Alma R. Toledo-Guillén, Alma R. Islas-Rubio

**Affiliations:** 1 Research Center for Food and Development, CIAD, Hermosillo, Sonora 83000, Mexico; E-Mails: claudiaberlanga@hotmail.com (C.M.B.-R.); arascon@ciad.mx (A.R.-C.); jalim@ciad.mx (J.L.-M.); atoledo@ciad.mx (A.R.T.-G.); aislas@ciad.mx (A.R.I.-R.); 2 Eastern Regional Research Center, Agricultural Research Service, US Department of Agriculture, 600 East Mermaid Lane, Wyndmoor, PA 19038, USA; E-Mails: kevin.hicks@ars.usda.gov (K.B.H.); madhav.yadav@ars.usda.gov (M.P.Y.)

**Keywords:** arabinoxylan, ferulic acid, protein, phase separation, controlled release

## Abstract

Wheat bran arabinoxylan (WBAX) gels entrapping standard model proteins at different mass ratios were formed. The entrapment of protein affected the gel elasticity and viscosity values, which decreased from 177 to 138 Pa. The presence of protein did not modify the covalent cross-links content of the gel. The distribution of protein through the network was investigated by confocal laser scanning microscopy. In mixed gels, protein aggregates forming clusters were detected at protein/polysaccharide ratios higher than 0.25. These clusters were not homogeneously distributed, suggesting that WBAX and protein are located in two different phases. The apparent diffusion coefficient (Dm) of proteins during release from mixed gels was investigated for mass ratios of 0.06 and 0.12. For insulin, Dm increased significantly from 2.64 × 10^−7^ to 3.20 × 10^−7^ cm^2^/s as the mass ratio augmented from 0.06 to 0.12. No significant difference was found for Dm values of ovalbumin and bovine serum albumin released from the mixed gels. The results indicate that homogeneous protein/WBAX gels can be formed at low mass ratios, allowing the estimation of Dm by using an analytical solution of the second Fick’s law.

## 1. Introduction

Gels are polymeric three-dimensional networks, which swell on contact with water but do not dissolve [1]. The water absorption property of gels confers on them interesting properties in applications such as food additives, enzyme immobilization and controlled release devices [2]. Although most studies are done on the synthetic polymers, gellable native or tailored polysaccharides (generally non-toxic and highly biocompatible), are receiving increasing attention [3,4]. Oral delivery of proteins remains to be a very active area of research as proteins are an important class of therapeutic agents and the oral route is the most widely accepted means of administration. However it is not feasible for direct delivery of protein drugs. To overcome the gastro-intestinal barriers various types of systems such as polymeric particles, and liposomes, among others, are investigated [4,5,6]. The colonic region, due to its lower proteolytic activity in comparison to that in the small intestine, has been considered as a possible absorption site for orally administrated peptides and proteins [7].

Arabinoxylans (AX) are non-starch polysaccharides isolated from the cell walls of cereal endosperm constituted by a linear backbone of β-(1→4)-linked xylose units containing α-l-arabinofuranosyl substituents attached through O-2 and/or O-3 [8,9,10,11]. Some of the arabinose residues of AX are ester-linked through (O)-5 to ferulic acid (FA) (3-methoxy, 4 hydroxy cinnamic acid), and so-called “ferulated AX”. Ferulated AX can make gels by covalent cross-linking of FA groups after oxidation by some chemical or enzymatic (laccase and peroxidase/H_2_O_2_ system) free radical-generating agents [12,13,14,15]. Diferulic acid (di-FA) and tri-ferulic acid (tri-FA) [16,17,18,19] have been identified as covalently cross-linked structures in AX gel. It has been reported that covalent bonds (di-FA and tri-FA) and physical interactions between AX chains contribute to the cross-linking process and define the gel characteristics [18,19,20]. Previous studies have indicated that AX gels present interesting properties such as neutral taste and odor, high water retention capacity (around 100 g water/gram AX) and stability under changes of pH or electrolytes. The meso and macroporous structure of AX gels with mesh sizes varying from 48 to 400 nm [20,21,22], and the dietary fiber nature of AX resistant to digestive enzymes but degraded by colonic microflora [7,8,9,10] qualify them for potential applications in colon-specific protein delivery. In this study, standard model proteins of different molecular weight were entrapped in AX gels at different protein/polysaccharide mass ratio and the distribution of protein through the network was investigated by confocal laser scanning microscopy (CLSM). The protein release capacity of mixed gels was then investigated.

## 2. Results and Discussion

### 2.1. WBAX and Protein/WBAX Gel

The gelation of WBAX solutions over time was rheologically investigated by a small amplitude oscillatory shear. For all samples, the storage (G') and loss (G'') modulus increased against time but with G' increasing faster than G''. At ~10 min, there was a cross-over where G' becomes greater than G'' which is associated with the gelation time. After 6 h of laccase exposure the G' and G'' values of WBAX gels were 177 and 20 Pa, respectively (Table 1), being close to those reported by Martínez-López *et al.* [22] in maize bran AX gels at 4% (*w*/*v*) (G' = 180 Pa, G'' = 2 Pa). The tan δ (G''/G') value at the end of gelation was 0.11, indicating the formation of a gel as according to Ross-Murphy [23] the gel point can be detected when G' becomes greater than G'' (*i.e.*, when tan δ becomes just less than 1). FA, di-FA and tri-FA contents of the WBAX measured before and after 6 h of gelation are given in Table 1. At the end of gelation, 70% of the FA initially present in WBAX was oxidized while only 60% was recovered as di and tri-FA. As a matter of fact, the di and tri-FA content in WBAX did not increase after laccase induced gelation, they rather decreased from 0.02 to 0.008 µg/mg polysaccharide. In a similar study, Lapierre *et al.* [24] and Carvajal-Millan *et al.* [25] reported that di-FA content in AX did not increase after gelation, in spite of a decrease in FA content. This behavior has been previously reported and related to the possible formation of higher ferulate crosslinking structures, which could be resistant to mild alkaline hydrolysis. Another explanation could be the contribution of residual lignin to the formation of AX polymer network [18,20,21,22]. In the present study, the predominant dimers in WBAX and WBAX/protein gels were 8-5', 5-5' and 8-*O*-4'. The 8-8' di-FA structure was not detected. These di-FA proportions are in agreement with previous reports on AX gels [18,19]. It has been proposed that the content and proportion of di-FA covalent bridges between AX chains could be involved in the final gel characteristics such as absence of pH, temperature or electrolyte susceptibility [11,22]. The swelling ratio (*q*, g water/g WBAX) at equilibrium swelling of WBAX gels in 15–20 h was 9, which is lower than the *q* value reported elsewhere by Berlanga-Reyes *et al.* [21] for 3.5% (*w*/*v*) maize bran AX gels induced by laccase (20 g water/g AX). The lower *q* value could be related to a more compact polymeric structure that limits the water absorption [26]. From swelling measurements, the molecular weight between two cross-links (*M*c), the cross-linking density (ρ_c_) and the mesh size (ξ) values of AX gels were calculated and found to be 29 × 10^3^ g/mol, 59 × 10^−6^ mol/cm^3^ and 57 nm, respectively (Table 1). These values were close to those reported in maize bran AX gels [22] induced by laccase (20 × 10^3^ g/mol, 75 × 10^−6^ mol/cm^3^ and 48 nm for *M*c, ρ_c_ and ξ, respectively). Higher ξ values (201–331 nm) have been reported in laccase-induced wheat flour AX gels at lower polysaccharide concentrations (0.5%–2% *w*/*v*) [20,27] and higher di-FA and tri-FA contents. The latter could be related to the high molecular weight reported in AX from wheat flour (438 kDa) [20] in comparison to the alkali-extracted AX from wheat bran (74 kDa) used in the present study. Lower ξ values in AX gels could be of interest as delivery systems for low molecular weight molecules.

Rheological tests and phenolic acids analysis were also performed in protein/WBAX gels (Table 2). The entrapment of protein in WBAX gels did not affect significantly the G' and G'' final values at protein/polysaccharide ratios of 0.06 and 0.12, which is in agreement with a previous report on gels at 0.04 protein/AX mass ratio [28]. At higher protein/WBAX mass ratios (0.25, 0.50 and 1.0), the addition of protein decreased the final G' value of the gel. At protein/WBAX ratio of 1, the G' value of the gel was diminished by 16%, 21% and 22% for insulin, ovalbumin and BSA, respectively, which are in the range previously reported at similar protein/AX mass ratio [29]. As presented in Table 2, for all protein/WBAX gels, phenolic acids content was not significantly affected, suggesting that the addition of protein decreases G' values, probably by affecting the physical interactions taking place between AX chains, as indicated by previous investigations [28,29].

**Table 1:** Characteristics of WBAX before and after 6 h gelation.

Characteristic	*t* = 0 h	*t* = 6 h
FA ^a^	0.020 ± 0.001	0.006 ± 0.001
di-FA ^a^	0.020 ± 0.001	0.008 ± 0.001
tri-FA ^a^	0.0005 ± 0.001	0.0003 ± 0.0001
G' ^b^	4	177 ± 3
G'' ^b^	6	20 ± 1
*M*c ^c^ × 10^3^	-	29 ± 1
ρ_c_^d^ × 10^−6^	-	59 ± 2
ξ ^e^	-	57 ± 8

^a^ Phenolic acids in µg/mg WBAX; ^b^ Storage (G') and loss (G'') modulus in Pa; ^c^ Molecular weight between two cross-links in g/mol; ^d^ Cross-linking density in mol/cm^3^; ^e^ Mesh size in nm. All values are means ± standard deviation of three repetitions.

**Table 2:** Characteristics of protein/WBAX mixtures after 6 h gelation.

Characteristic	Insulin/WBAX Mass Ratio				
	**0.06**	**0.12**	**0.25**	**0.50**	**1.0**
FA ^a^	0.005 ± 0.001	0.006 ± 0.001	0.005 ± 0.001	0.006 ± 0.001	0.005 ± 0.001
di-FA ^a^	0.007 ± 0.001	0.008 ± 0.001	0.007 ± 0.001	0.007 ± 0.001	0.007 ± 0.001
tri-FA ^a^	0.0002 ± 0.0001	0.0003 ± 0.0001	0.0002 ± 0.0001	0.0002 ± 0.0001	0.0002 ± 0.0001
G' ^b^	178 ± 4	176 ± 4	165 ± 3	155 ± 3	145 ± 4
G'' ^b^	21 ± 2	20 ± 2	19 ± 2	18 ± 3	16 ± 2
	**Ovalbumin/WBAX Mass Ratio**				
	**0.06**	**0.12**	**0.25**	**0.50**	**1.0**
FA ^a^	0.005 ± 0.001	0.005 ± 0.001	0.005 ± 0.001	0.006 ± 0.001	0.006 ± 0.001
di-FA ^a^	0.007 ± 0.001	0.006 ± 0.001	0.007 ± 0.001	0.007 ± 0.001	0.006 ± 0.001
tri-FA ^a^	0.0003 ± 0.0001	0.0003 ± 0.0001	0.0002 ± 0.0001	0.0003 ± 0.0001	0.0002 ± 0.0001
G' ^b^	176 ± 4	177 ± 3	160 ± 3	158 ± 3	140 ± 3
G'' ^b^	20 ± 2	20 ± 3	19 ± 2	17 ± 3	17 ± 2
	**BSA/WBAX Mass Ratio**				
	**0.06**	**0.12**	**0.25**	**0.50**	**1.0**
FA ^a^	0.006 ± 0.001	0.006 ± 0.001	0.006 ± 0.001	0.005 ± 0.001	0.006 ± 0.001
di-FA ^a^	0.007 ± 0.001	0.006 ± 0.001	0.008 ± 0.001	0.006 ± 0.001	0.007 ± 0.001
tri-FA ^a^	0.0002 ± 0.0001	0.0002 ± 0.0001	0.0002 ± 0.0001	0.0002 ± 0.0001	0.0003 ± 0.0001
G' ^b^	176 ± 5	174 ± 5	165± 3	155 ± 4	138 ± 5
G'' ^b^	20 ± 2	21 ± 3	18 ± 2	16 ± 3	15 ± 2

^a^ Phenolic acids in µg/mg WBAX; ^b^ Storage (G') and loss (G'') modulus in Pa. All values are means ± standard deviation of three repetitions.

### 2.2. Protein Distribution in the Gels

**Figure 1:**
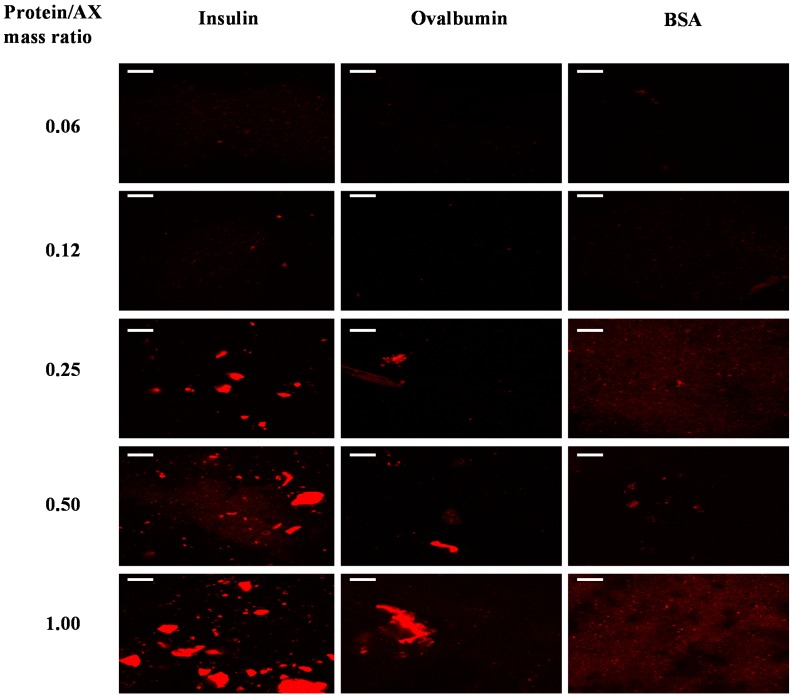
Confocal laser scanning microscopy (CLSM) observations of WBAX gels at different protein/polysaccharide mass ratios. Proteins stained by rhodamine isothiocyanate (RITC) at 0.02% (*w*/*v*). Protein phase is bright zone (scale bar = 20 µm).

The distribution of protein through the network of the mixed protein/WBAX gels was investigated by a CLSM study. The bright zones corresponding to protein aggregates forming clusters were detected at protein/polysaccharide ratios of 0.25, 5.0 and 1.0. The size of protein aggregate increased with the increased amount of entrapped protein (Figure 1). These clusters were not homogenously distributed, suggesting that polysaccharide and protein are located in two different phases as a result of thermodynamic incompatibility. A similar distribution pattern was observed for all three standard proteins used in this study: insulin, ovalbumin, and bovine serum albumin (BSA). It has been reported that in neutral polysaccharide and protein mixture, the protein tend to form aggregates and the mixed biopolymers become incompatible at higher concentration leading to a segregated phase separation [30].

### 2.3. Protein Release

**Figure 2:**
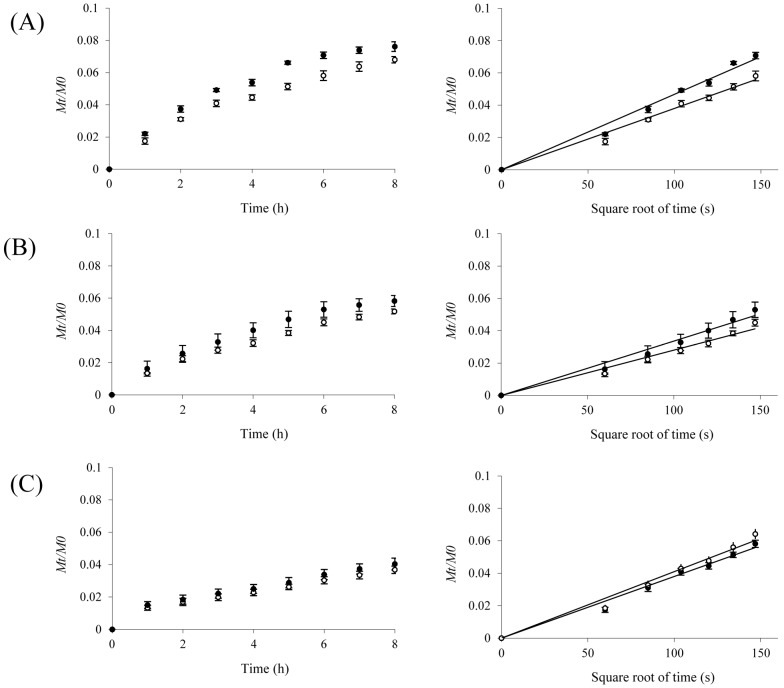
Cumulative release (*M_t_*/*M*_0_) of insulin (**A**), ovalbumin (**B**) and bovine serum albumin (**C**) from wheat bran arabinoxylan (WBAX) gels as a function of time and as a function of root time. Protein release was followed at 25 °C and 90 rpm during 8 h. Gels at protein/WBAX mass ratios of 0.06 (Ο) and 0.12 (●).

The release of proteins from gels was investigated for mass ratios of 0.06 and 0.12, since those ratios showed homogenous distribution of protein through the network (explained in the Section 2.2), allowing the estimation of the apparent diffusion coefficient (Dm) by using an analytical solution of the second Fick’s law. Figure 2 presents the protein release profiles of standard proteins, at given protein/WBAX mass ratio. At the same mass ratio, a different release rate was obtained for the three proteins tested. As shown in Figure 2, a linear relationship between the cumulative release (*M_t_*/*M*_0_) of proteins and the square root of time was found for protein release from gels, which allowed us to calculate Dm as given in Table 2. The diffusion coefficients of these proteins in water (Do) as reported in the literature [31,32,33] are also included for comparison. For insulin, Dm increased significantly from 2.64 × 10^−7^ to 3.20 × 10^−7^ cm^2^/s as the mass ratio augmented from 0.06 to 0.12 but no significant difference was found for Dm values of ovalbumin and BSA. The similar Dm values found for ovalbumin or BSA at 0.06 and 0.12 mass ratio could be related to a possible pore size obstruction effect since WBAX gels would present a heterogeneous structure due to the movement of WBAX chains in the network, which could decrease the network free space for the protein transport [27]. The Dm values found in the present study were in the range reported by other researchers for proteins in hydrated gels [34]. The entrapment and release of insulin in maize bran AX gels at 2.5% (*w*/*v*) has been investigated and reported previously (protein/AX mass ratio of 0.04, G' value of 9 Pa, mesh size of 80 nm and di-FA and tri-FA content of 0.03 and 0.015 µg/mg AX, respectively) [35]. That study reported an insulin Dm value of 0.99 × 10^−7^ cm^2^/s, which is lower than the one obtained in the present research (2.64 × 10^−7^ to 3.20 × 10^−7^ cm^2^/s). The lower Dm could be due to the lower insulin/AX mass ratio used in the previous study (0.04) in comparison to the present study (0.06 and 0.12). A higher protein amount entrapped in the gel can induce a loss in connectivity of the AX network and/or an increase of protein gradient concentration between the gel and the recovery medium [30]. A method where different proteins are loaded into cured wheat AX gels by allowing protein solution to diffuse into the gel, has been reported; however, this method wastes protein and requires long loading times [27]. With protein/AX mass ratios from 0.1–0.4, these authors found Dm values from 3.0 × 10^−7^ to 6.8 × 10^−7^ cm^2^/s and from 1.9 × 10^−7^ to 5.9 × 10^−7^ cm^2^/s for ovalbumin and BSA, respectively, which are higher than those observed in the present research for the same proteins. These differences in Dm could be related to the higher mesh size values of WEAX gels used in that study (201–331 nm) in comparison to our current study (58 nm). The differences in Dm values for the model proteins tested in the different protein/WBAX gels were also reflected in the percentage of protein released at the end of gel incubation periods. The percentages of protein released were from 3.2–7.5 (Table 3). In general, a low amount of the entrapped protein was released by diffusion. This result indicates that as delivery matrices, protein/WBAX gels would liberate most of entrapped protein only after gel degradation in specific sites such as the colon where AX can be degradated by colonic bacteria. These results become of importance when it is desirable to protect a carried therapeutic protein or peptide (for example insulin) from the gastric environment for further release in the colon region after gel degradation by colonic bacteria. Previous reports indicate the absorption of protein or peptide drugs in the colonic region [36]. In addition, it has been previously reported that cross-linked AX can be fermented by colonic bacteria [7,8,9,10].

**Table 3:** Apparent diffusion coefficients and release of proteins with different molecular weight in wheat bran arabinoxylan (WBAX) gels at different protein/polysaccharide mass ratios.

Protein	*M*_W_^a^ (kDa)	Do ^b^ × 10^−7^ (cm^2^/s)	Dm ^c^ × 10^−7^ (cm^2^/s)		Protein Released (%)	
			Protein/WBAX Mass Ratio		Protein/WBAX Mass Ratio	
			0.06	0.12	0.06	0.12
Insulin	5	15.9	2.64 ± 0.07	3.20 ± 0.06	6.2 ± 0.4	7.5 ± 0.5
Ovalbumin	43	8.40	1.47 ± 0.01	1.49 ± 0.04	5.1 ± 0.5	4.4 ± 0.3
BSA ^d^	67	6.80	1.20 ± 0.01	1.25 ± 0.03	3.5 ± 0.2	3.2 ± 0.3

^a^ Molecular weight of proteins; ^b^ Diffusion coefficient of proteins in water from the literature [29,30,31]; ^c^ Apparent diffusion coefficient of proteins in the WBAX gels; ^d^ Bovine serum albumin. All values are average from two repetitions.

## 3. Experimental Section

### 3.1. Materials

WBAX were extracted from wheat bran as previously described by Berlanga-Reyes *et al.* [26]. WBAX presented an arabinose-to-xylose (A/X) ratio of 0.80, a FA content of 0.008 µg/mg arabinoxylans, a molecular weight of 74 kDa, and a [η] of 206 mL/g. Laccase (benzenediol:oxygen oxidoreductase, E.C.1.10.3.2) from *Trametes versicolor*, and all chemical reagents were from Sigma Chemical Co. (St. Louis, MO, USA). Laccase activity was measured as reported elsewhere [19].

### 3.2. Preparation of WBAX Gels

An WBAX solution (5% *w*/*v*) was prepared in citrate phosphate buffer 0.1 M pH 5.5. This solution was mixed with laccase (1.675 nkat/mg WBAX). Gels were allowed to form for 6 h at 25 °C.

### 3.3. Phenolic Acids Content

FA, di-FA and tri-FA contents were determined by reversed phase high performance liquid chromatography (RP-HPLC) after their de-esterification from AX gels. An Alliance Waters e2695 separation module with a Waters 2998 photodiode array detector (Waters, Milford, MA, USA) was used. To 2 mL of WBAX or protein/WBAX gel, 2 mL of 2 N NaOH was added and the mixture was allowed to react for 1 h in the dark at 20 °C under argon. After adding 3,4,5-trimethoxy-*trans*-cinnamic acid (TMCA, internal standard, 5 µg), the pH was adjusted to 2.0 ± 0.02 with 2 N HCl. Phenolic were extracted twice with diethyl ether and evaporated at 30 °C under argon. The dried extracts were solubilized in 0.5 mL of methanol, filtered (0.45 µm) and injected (20 µL) into RP-HPLC as previously described [18,26]. Response factors of di-FA and tri-FA determined by Saulnier *et al.* [36] and Rouau *et al.* [37], respectively were used.

### 3.4. Rheological Tests

Rheological tests were performed by small amplitude oscillatory shear by using a strain controlled rheometer (ARES 2000, Rheometric Expansion System, Rheometric Scientific, Champ sur Marne, France) as reported before [18,21]. WBAX and protein/WBAX gelation was studied for 6 h at 25 °C. All measurements were carried out at 0.25 Hz frequency and 5% strain (in linear domain).

### 3.5. WBAX Gel Swelling

WBAX gels were allowed to swell in 20 mL of 0.02% *w*/*v* sodium azide solution as previously described [26]. The swelling ratio (*q*) was calculated as:

(1) $$q=\left(W_{\text{s}}-W_{\text{d}}\right)/W_{\text{d}}$$

where *W*_S_ is the weight of swollen gels and *W*_d_ is the weight of WBAX in the gel [20].

### 3.6. WBAX Gel Structure

From swelling measurements, the molecular weight between two cross-links (*M*c) was calculated using the classic Flory and Rehner [38,39] modified by Peppas and Merrill [40] analysis for gels where the cross-links are introduced in solution.

(2) 1Mc=2Mn−(vV1)(ln(1−v2,s)+v2,s+x1(v2,s)2v2,r[(v2,sv2,r)13−12(v2,sv2,r)]

In this equation, *M*n is the number average molecular weight of WBAX (only the xylose backbone was considered). The *M*n value of WBAX was calculated from their [η] values by using the Mark–Houwink equation as reported by Carvajal-Millan *et al.* [20]. *V*_1_ is the molar volume of water (18 cm^3^/g), *v*_2,r_ and *v*_2,s_ represent the values of polymer volume fraction inside the gel, in a relaxed state (*v*_2,r_) and at equilibrium swelling (*v*_2,s_). *x*_1_ is the Flory polymer-solvent interaction parameter. The arabinoxylans-water system was calculated to correspond to theta conditions and was therefore taken as equal to 0.5 for the present system [20]. The partial specific volume (*v*) of WBAX was 0.59 cm^3^/g. After *M*c calculation the average mesh size (ξ) and the cross-linking density (ρ_c_) in the WBAX gels were calculated as reported by Carvajal-Millan *et al.* [20].

(3) $$\text{ξ}={v_{2,\text{s}}}^{-1/3}{\left(2C_{n}M\text{c}/M\text{r}\right)}^{1/2}l$$

(4) $${\text{ρ}}_{\text{c}}=1/vM\text{c}$$

In Equation (3) *M*r represents the molecular weight of the repeating unit (xylose, 132 g/mol), *C_n_* the characteristic ratio for arabinoxylans (11.5) and *l* the bond length between two xyloses (0.286 nm).

### 3.7. Preparation of Protein/WBAX Gels

WBAX and insulin (5 kDa), ovalbumin (43 kDa) or BSA (67 kDa) solutions in 0.05 M citrate phosphate buffer pH = 5.5 were mixed in order to prepare five protein/WBAX mixtures at mass ratios of 0.06, 0.12, 0.25, 0.5 and 1.0. Laccase (1.675 nkat per mg WBAX) was added as cross-linking agent. Gels were allowed to form for 6 h at 25 °C.

### 3.8. CLSM

CLSM (Leica Microsystems, Mannheim, Germany) was used in the fluorescent mode, with rhodamine isothiocyanate (RITC) at 0.02% (*w*/*v*) as fluorescent probe as described previously [41].

### 3.9. Protein Release

Protein release from protein/WBAX gels was studied according to the procedure of Carvajal-Millan *et al.* [27]. Two milliliters of protein/WBAX mixtures were poured into a 30 mL cylindrical plastic cell (30 mm diameter) just after laccase addition (1.675 nkat per mg WBAX). Protein/WBAX gels were allowed to form for 6 h at 25 °C. Then, protein was released in 6 mL of distilled water placed on the gel surface. Sodium azide (0.02% *w*/*v*) was added to the liquid medium to prevent microbial contamination. Protein release was performed at 25 °C and 90 rpm tangential rotation during 24 h. Sodium azide was replaced at every sampling hour. At each time, 1 mL was taken for quantification of released protein by Bradford’s assay [42]. At the end of the test, the protein/WBAX gels were hydrolyzed by xylanase (100 µL; 15 nkat/µL) in order to quantify the un-released protein. Xylanase was added to the gel surface and incubated 2 h at 25 °C and 90 rpm tangential rotation. Protein recovery (released protein + un-released protein) reached almost 100%. Protein release from protein/WBAX gels was characterized by calculating an apparent diffusion coefficient (Dm). This Dm was estimated from the release kinetics curve, fitted by using an analytical solution of the second Fick’s law, which gives the solute concentration variation as a function of time and distance [42].

(5) $$M_{t}/M_{0}=\left(4/L\right){\left(\text{Dm}t/\text{π}\right)}^{0.5}$$

where *M_t_* is the accumulated mass of protein released at time *t*, *M*_0_ is the mass of protein in the gel at time zero, *L* is the sample thickness (0.3 cm) and Dm is the apparent diffusion coefficient. By plotting the relative solute mass released (*M_t_*/*M*_0_) at time *t*, *versus* the square root of time, a simplified determination of Dm can be made assuming that Dm is constant and that the sample is a plate with a thickness (*L*). In this study the apparent Dm was calculated from the linear part of the (*M_t_*/*M*_0_) (*t*) curves. The percentage of protein released at the end of the test was also calculated.

## 4. Conclusions

The standard proteins used as a model in this study did not have any negative effect on the rheological and microstructural characteristics of protein/WBAX gels at mass ratios of 0.06 and 0.12. Nevertheless, at higher mass ratios (0.25, 0.5 and 1.0) the storage modulus of the mixed gels decreased by 20%, presenting proteins and polysaccharide located in two different phases. The presence of protein did not modify the covalent cross-links content of the mixed gels. For a low molecular weight protein such as insulin, release rate and quantity can be significantly modified by varying the amount of protein entrapped. Only a low amount of the protein entrapped in WBAX gels is released by diffusion. These results indicate that homogeneous protein/WBAX gels can be formed at low mass ratios, allowing the estimation of Dm by using an analytical solution of the second Fick’s law. Complementary research is in progress in order to evaluate these protein/WBAX gels under simulated gastrointestinal conditions.
